# Retraction: Avola, R. et al. Blue Light Induces Down-Regulation of Aquaporin 1, 3, and 9 in Human Keratinocytes. *Cells* 2018, *7*, 197

**DOI:** 10.3390/cells8080819

**Published:** 2019-08-03

**Authors:** Cells Editorial Office

**Affiliations:** MDPI, St. Alban-Anlage 66, 4052 Basel, Switzerland; cells@mdpi.com

It has come to our attention that the Western blot data shown in Figure 3 of [1] had been heavily edited and did not show the original data in a complete way. We requested the authors to supply the original gel data, which was examined by experts from our Editorial Board. They concluded that the full images showed an absence of the AQP3, which should have been glycosylated and present in the gel. Since real-time PCR data was not presented in the paper, it was concluded that the results of the paper are no longer sufficiently supported. The published paper [1] will therefore be marked as retracted.

MDPI is a member of the Committee on Publication Ethics and takes seriously its responsibility to publish only high-quality research. We regret that these issues were not identified earlier and apologize to readers of the journal. We note that this action is not supported by the authors, who maintain that the original results still stand.
